# Bone health assessment of patients with juvenile idiopathic arthritis: a comparison between DXA and bonexpert

**DOI:** 10.1186/1546-0096-12-S1-P17

**Published:** 2014-09-17

**Authors:** Charlotte M Nusman, Janneke Anink, Marieke Otten, Marion van Rossum, Rick van Rijn, Mario Maas, Lisette van Suijlekom-Smit

**Affiliations:** 1 Radiology, Academic Medical Center, Netherlands; 2 Pediatric Hematology, Immunology, Rheumatology and Infectious Disease, Emma Children's Hospital , Amsterdam, Netherlands; 3 Pediatrics/Pediatric Rheumatology, Sophia Children's Hospital, Erasmus MC, Rotterdam, Netherlands; 4 Pediatric Rheumatology, Reade Institute, location Jan van Breemen, Netherlands; 5 Pediatrics, Emma Children's Hospital AMC, Amsterdam, Netherlands

## Introduction

Juvenile idiopathic arthritis (JIA) affects bone mineral density (BMD) due to chronic inflammation, glucocorticoid treatment and immobilization. Dual-energy X-ray absorptiometry (DXA) is most widely used to determine BMD. BoneXpert is a new, feasible and reproducible method for automatic determination of cortical BMD on hand radiographs. Moreover radiation exposure is low and in low-risk peripheral areas.

## Objectives

The aim of this study is to compare BoneXpert and DXA in the assessment of BMD in JIA patients.

## Methods

Thirty-five JIA patients with available DXA and hand radiograph within the same time period were included from a tertiary hospital of the Dutch Arthritis and Biologicals in Children register. Outcome measures for BMD were Bone Health Index (BHI) from BoneXpert and BMD total body, BMD lumbar spine and Bone Mineral Apparent Density (BMAD) from DXA. For all outcome measures Z-scores were calculated. Correlations between BMD measurements by DXA and BoneXpert were assessed with Pearson correlation coefficients.

## Results

The patients in this study had significantly lower mean BMD compared to the healthy population on all BMD measures (p<0.05). The pearson correlation coefficient for the absolute scores of DXA BMD and BHI varied between 0.568-0.770 (p=0.000). The correlation coefficient for the Z-scores of DXA and BoneXpert (0.127-0.322) was not significant.

## Conclusion

BHI measured by BoneXpert is correlated to measurements of BMD by DXA. The correlation of Z-scores of BMD measured by the two methods is weaker. Longitudinal studies and assessment of the association of the BMD measurements with outcome (for instance atraumatic fractures) are necessary to determine the value of BoneXpert in clinical use.

## Disclosure of interest

None declared.

